# Characterization of *Abcc4* Gene Amplification in Stepwise-Selected Mouse J774 Macrophages Resistant to the Topoisomerase II Inhibitor Ciprofloxacin

**DOI:** 10.1371/journal.pone.0028368

**Published:** 2011-12-05

**Authors:** Béatrice Marquez, Geneviève Ameye, Coralie M. Vallet, Paul M. Tulkens, Hélène A. Poirel, Françoise Van Bambeke

**Affiliations:** 1 Université catholique de Louvain, Louvain Drug Research Institute, Pharmacologie cellulaire et moléculaire, Brussels, Belgium; 2 Université catholique de Louvain, Cliniques universitaires Saint-Luc, Centre de Génétique humaine, Brussels, Belgium; University of Cambridge, United Kingdom

## Abstract

Exposure of J774 mouse macrophages to stepwise increasing concentrations of ciprofloxacin, an antibiotic inhibiting bacterial topoisomerases, selects for resistant cells that overexpress the efflux transporter Abcc4 (Marquez et al. [2009] *Antimicrob. Agents Chemother.* 53: 2410–2416), encoded by the *Abcc4* gene located on Chromosome 14qE4. In this study, we report the genomic alterations occurring along the selection process. Abcc4 expression progressively increased upon selection rounds, with exponential changes observed between cells exposed to 150 and 200 µM of ciprofloxacin, accompanied by a commensurate decrease in ciprofloxacin accumulation. Molecular cytogenetics experiments showed that this overexpression is linked to *Abcc4* gene overrepresentation, grading from a partial trisomy of Chr 14 at the first step of selection (cells exposed to 100 µM ciprofloxacin), to low-level amplifications (around three copies) of *Abcc4* locus on 1 or 2 Chr 14 (cells exposed to 150 µM ciprofloxacin), followed by high-level amplification *of Abcc4* as homogeneous staining region (hsr), inserted on 3 different derivative Chromosomes (cells exposed to 200 µM ciprofloxacin). In revertant cells obtained after more than 60 passages of culture without drug, the *Abcc4* hsr amplification was lost in approx. 70% of the population. These data suggest that exposing cells to sufficient concentrations of an antibiotic with low affinity for eukaryotic topoisomerases can cause major genomic alterations that may lead to the overexpression of the transporter responsible for its efflux. Gene amplification appears therefore as a mechanism of resistance that can be triggered by non-anticancer agents but contribute to cross-resistance, and is partially and slowly reversible.

## Introduction

Overexpression of multidrug transporters (MDR) from the ATP-binding cassette (ABC) family is now widely recognized as a mechanism of resistance to cytotoxic drugs and is associated with therapeutic failures in patients receiving anticancer chemotherapy [1]. Several mechanisms have been described as leading to the overexpression of multidrug transporters, like induction of gene transcription (possibly caused by the drug itself ([2] for review)), increase in mRNA stability [3], epigenetic changes [4], [5], or gene amplification [6]. These mechanisms have been explored so far mainly in hepatocytes exposed to different xenobiotics, where activation of gene transcription by nuclear receptors has been well documented [7]. In cells exposed to anticancer agents, chromosomal alterations have also been reported after selection *in vivo* [8] or *in vitro* upon chronic exposure to drugs [9], but the underlying mechanisms can be much more diverse (see for a few examples [10]–[12]).

By its efflux properties, the multidrug transporter ABCC4 (MRP4) protects cells against toxicity induced by antimetabolites, such as methotrexate or analogues of purines and nucleosides, or by type I topoisomerase inhibitors, such as camptothecins [13]–[15]. ABCC4 overexpression has been reported in cancer cells, such as in prostate tumors [16] or human leukemic cells (with *in vitro* acquired resistance to 6-mercaptopurine [17]), and is associated with a poor clinical outcome in neuroblastoma [18]. Moreover, single nucleotide polymorphisms in *ABCC4* gene have been shown to modulate the therapeutic response to methotrexate in children suffering from acute lymphoblastic leukemia [19].

Because of their broad substrate specificity, multidrug transporters can also reduce the cellular accumulation of other drugs and impair their activity if their pharmacological target is intracellular [2]. Conversely, these drugs can also trigger the overexpression of their transporters, as demonstrated for ABCC4 with the antiviral agent adefovir [20] and the fluoroquinolone antibiotic ciprofloxacin [21]. Fluoroquinolones are potent and widely used antibacterial agents that show a marked accumulation in eukaryotic cells, which explains their activity against a large array of intracellular bacteria (see [22] for review). Fluoroquinolones act by inhibiting the prokaryotic type II topoisomerase enzymes (DNA gyrase and topoisomerase IV). Although 100 to 1000-fold more active against bacterial enzymes than against their mammalian homologue topoisomerase II [23], fluoroquinolones can also cause genotoxic and clastogenic effects in eukaryotic cells at high concentrations [24], which has raised concerns about potential toxicities if used at supratherapeutic concentrations [25].

Applying to J774 macrophages a method widely used *in vitro* to select tumor cell lines resistant to anticancer drugs [26] and which consists in exposing cells to progressively increasing concentrations of the drug of interest, we were able to select, after about 50 passages in the presence of ciprofloxacin, cell lines in which the accumulation of this fluoroquinolone was markedly reduced [27]. This phenotype is associated with an accelerated efflux of ciprofloxacin that has been ascribed to an increased expression of *Abcc4* (*Mrp4*) mRNA [21]. We also showed that Abcc4 protein overexpression was only slowly reversible, as more than 60 passages in the absence of ciprofloxacin were needed to obtain cells displaying a phenotype similar to that of the wild-type cell line (similar level of ciprofloxacin accumulation [27] despite a residual slight increase in Abcc4 protein content [21]). All together these data suggested that overexpression of *Abcc4* could be driven through gene amplification.

The present study therefore focuses on the characterization of the progressive acquisition of multidrug resistance in J774 macrophages collected along the selection process with ciprofloxacin and examines possible genomic amplification of *Abcc4* in these cells by fluorescence in situ hybridization (FISH) and multicolor FISH (mFISH). Our data show that *Abcc4* gene amplification indeed occurs in the resistant cells and that it is slowly reversible. We also evidence other clonal chromosomal alterations developing along the selection process, which may reflect the genomic instability induced in eukaryotic cells when exposed to high concentrations of ciprofloxacin.

## Results

### Characterization of ciprofloxacin accumulation and Abcc4 expression in cell lines resistant to different concentrations of ciprofloxacin

We showed previously that mouse J774 macrophages resistant to 200 µM ciprofloxacin were characterized by a markedly reduced accumulation of this drug [27] that was attributed to the overexpression of the ABC transporter Abcc4 [21].

**Figure 1:**
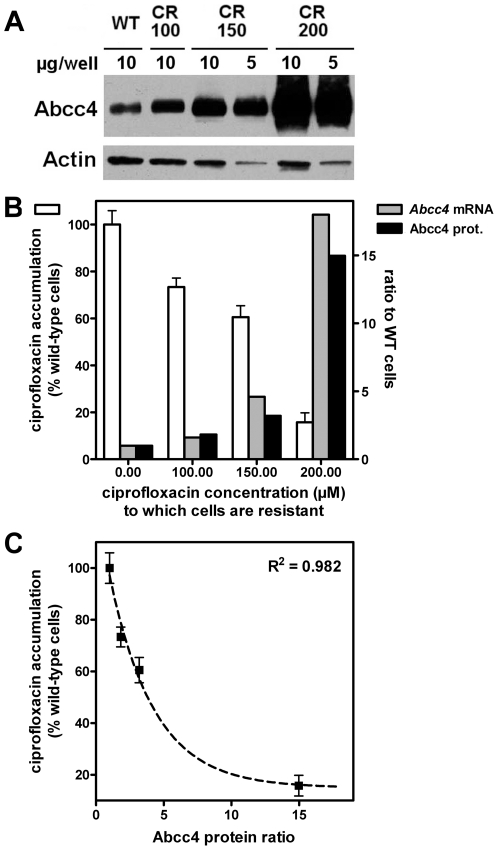
Relationship between cellular accumulation of ciprofloxacin and Abcc4 expression. A. Upper panel: western blot of Abcc4 (and actin as loading control) in cell lysates from wild-type (WT) macrophages and from cells resistant to different concentrations of ciprofloxacin (100 µM [CR100], 150 µM [CR150] and 200 µM [CR200]). B. Ciprofloxacin accumulation and Abcc4 mRNA and protein relative expression in cells made resistant to increasing concentrations of ciprofloxacin; (i) left axis (open bars): accumulation of ciprofloxacin in % (mean ± SD [n = 3]) of the value measured in wild-type cells incubated during 2 h with 50 µM ciprofloxacin [absolute value: 162 ng/mg prot.]); (ii) right axis: Abcc4 mRNA (grey bars) and protein (black bars) levels as a ratio to the value observed in wild-type cells (set to 1). C. Correlation between ciprofloxacin residual accumulation and Abcc4 relative expression in these cells. The curve corresponds to a best fit based on an inverse logarithmic function (y = 11.5×e^−**0.3037 X**^+14.92).

To further understand the mechanisms leading to this overexpression, we have now compared the accumulation of ciprofloxacin and the expression of Abcc4 (protein and mRNA levels) in wild-type cells *vs.* cells resistant to ciprofloxacin concentrations of 100, 150 and 200 µM. The data illustrated in Figure 1 show that the accumulation of ciprofloxacin was decreased in parallel with the increased level of resistance. However, the process was not linearly related to the drug concentration used for selection, most of the effect being obtained only in cells resistant to the highest concentration. This reduction of ciprofloxacin accumulation was associated with a commensurate increase in the expression of Abcc4, both at the mRNA and protein levels. The lowest panel of Figure 1 shows the correlation between ciprofloxacin accumulation and protein levels of Abcc4.

### Characterization of the *Abcc4* locus amplification in ciprofloxacin-resistant and revertant cell lines compared to wild-type macrophages

**Figure 2:**
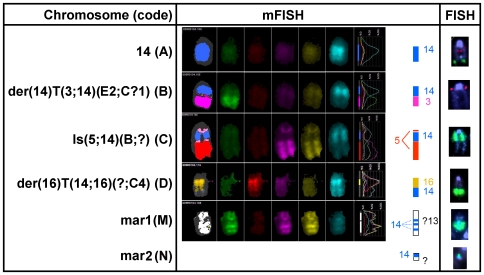
Molecular cytogenetics of relevant chromosomes in wild-type, fully ciprofloxacin-resistant, and revertant macrophages. mFISH and FISH experiments (red: control probe; green: *Abcc4* probe) for relevant chromosomes in wild-type macrophages, in the clones of the fully ciprofloxacin-resistant macrophages CR200, and in the revertant macrophages (Rev200). Karyotypes of the cell lines (abnormalities illustrated in the figure are highlighted in bold): a) wild-type cells: 72<3n>,X,der(X)T(X;11)(E or F1;?B5),der(1)T(1;6)(C?;B?3), +der(1)(1A1→1C?::6B?3→6D∼F::X? →X?),−3,+der(5;17)(5A1→5C2::17A1→17?), der(6)T(1;6)(?D;B3),+der(6),+der(9)T(9;19)(?B;C2),+?Del(12)(?B),−13,idic(13), **der(14)T(3;14)(E2;C?1)**,idic(15),−18,−19,idic(19),ace(3)x2,ace(18)x2; b) CR200 cells (“idem” refers to the chromosomal abnormalities stated in the wild-type cells karyotype): – clone I: 72,idem,**Is(5;14)(B;?)**,+9,−der(9)T(9;19),+13,−idic(13),ace(3)x2∼3, ace(?14)x0∼2,ace(18)x1∼2[cp17]; – clone II: 72,idem,?+9,−der(9)T(9;19),+13,−idic(13),**mar1x2∼3**, ace(?14)x1∼2,ace(18)x1∼2[cp3]; – clone III: 71,idem,+der(1)T(1;2)(H?;?),−der(9)T(9;19),+13, −idic(13),**der(16)T(14;16)(?;C4)**,ace(3)x3,ace(?14)x0∼2[cp2].

**Figure 3:**
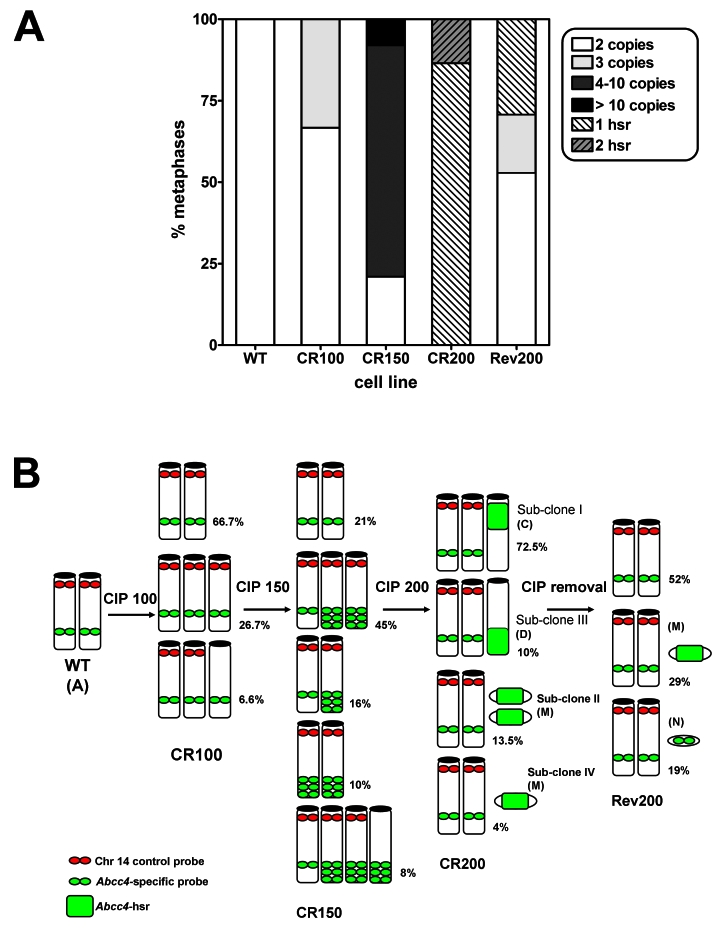
*Abcc4* copies detected by FISH on metaphases of cultured macrophage cell lines. A. Relative abundance of the clones according to number of *Abcc4* copies detected by FISH in the different cell lines (WT: wild-type cells; CR100, 150, and 200: cells resistant to 100, 150, and 200 µM of ciprofloxacin; Rev200: revertant cells). Hsr, homogeneous staining region. B. Schematic representation of chromosomes with *Abcc4* copies in the same cell lines (only chromosomes with *Abcc4* copies are shown; Chr der(14)T(3;14)(E2;C?1) (Chr B), present in almost all clones, but which lacks *Abcc4* locus, is not represented). Percentages refer to the relative abundance of each clone, and letters between brackets to Chromosomes as identified in Figure 2.

Conventional karyotype coupled to Multicolor FISH (mFISH) showed that the wild-type J774 cell line was characterized by a near-triploid karyotype (Figure S1) and exhibited particularly a derivative of Chr 14 characterized by the replacement of its telomeric end by a part of Chr 3 (Figure 2, Chr B) in addition to the two apparently normal Chromosomes 14. The *Abcc4* locus localized at Chr 14qE4 was analyzed by FISH using BAC (Bacterial Artificial Chromosome) probes, on metaphases in comparison to a more centromeric control locus (see Figure S2 for typical images and Figure 3 for a schematic representation of chromosomes with *Abcc4* loci observed in the different cell lines and a quantification of the different clones).

Wild-type macrophages remained disomic for *Abcc4* locus, as we observed 2 different copies of *Abcc4* on two apparently normal Chromosomes 14 (Figure 2, Chr A), in comparison to the control probe (normally located at Chr 14qA3), which exhibited a third signal on the derivative Chromosome 14 (Figure 2, Chr B). In the intermediate resistant cell line CR100, we predominantly observed the wild-type pattern (almost 67% of the metaphases). A third copy of the *Abcc4* locus was identified in about 1/3 of the cells, either on an additional apparently normal Chromosome 14 with both *Abcc4* and control probes (27% of the metaphases, Figure 3), or on a marker Chromosome (7% of the metaphases). At the next step of selection with ciprofloxacin (CR150 cells), only 21% of the analyzed metaphases displayed the wild-type pattern while all the others showed different clones characterized by a low level chromosomal amplification of *Abcc4* (approximately 3 copies) located either on one or on two Chromosomes 14, or associated to a marker Chromosome which did not exhibit the Chromosome 14 centromeric control probe.

In the fully ciprofloxacin-resistant cell line CR200, we observed *Abcc4* high-level amplification as a homogenous staining region (hsr) in all metaphases (Figure S2) on different marker Chromosomes that did not exhibit the Chromosome 14 centromeric control probe. Of note, all metaphases observed for the CR200 cell line displayed 2 apparently normal Chr 14 and no *Abcc4* copy on the derivative Chr 14, as in wild-type cells. To identify the marker Chromosomes with *Abcc4* hsr, we performed a mFISH analysis followed by *Abcc4* hybridization (Figure 2 and Figure S2). Upon examination of 73 metaphases, the hsr was found with a localization that was either centromeric to a derivative Chr 5 (72.5% of the metaphases, clone I) (Figure 2, Chr C) or telomeric to a derivative Chr 16 (10%, clone III) (Figure 2, Chr D). Interestingly, in 13.5% and 4% of the observed metaphases, the *Abcc4* amplification was found on either two (clone II) or one (clone IV) smaller marker Chromosome(s) (Figure 2, Chr M). These contained three heterochromatic regions aside of the centromere and were suspected to correspond to a derivative of Chr 13 by mFISH, although the chromosomal origin of the centromeric region remained uncertain (Figure 2, Chr M). Other additional genomic alterations were acquired in the resistant cells, namely −der(9)T(9;19),+13,−idic(13) in all clones, +9, in clones I and II, and +der(1)T(1;2)(H?;?) in clone III, as identified by mFISH.

In the revertant cell line Rev200 for which 31 metaphases were examined, we observed the wild-type phenotype (2 apparently normal Chr 14, and one without the *Abcc4* locus) in around half of them (16), but 9 metaphases displayed a remaining high-level amplification of *Abcc4* on the small marker Chr M (Figure 3), as already observed in the CR200 cell line. Moreover, in the remaining 6 metaphases, we observed an additional copy of *Abcc4*, located on a very small unidentified marker Chromosome (Chr N; Figures 2 and S2). All these observations were in agreement with the analysis of interphasic cells, made on a larger cell population (200 nuclei), for each cell line (data not shown).

### Characterization of the *DnajC3* locus amplification in resistant cell lines

**Figure 4:**
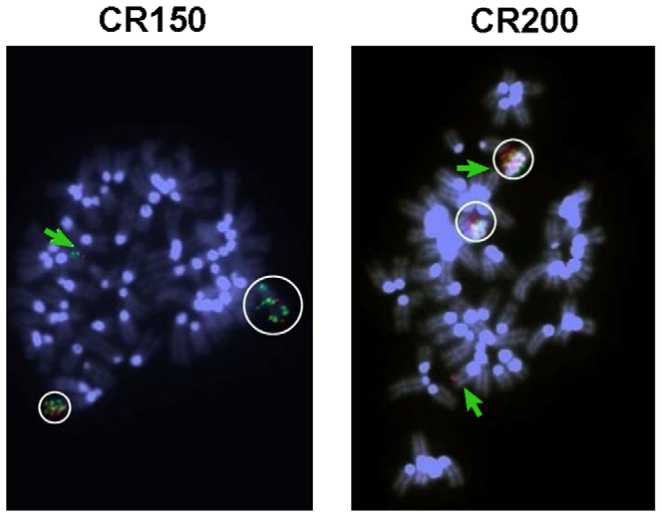
*DnajC3* and *Abcc4* FISH analysis in selected resistant cell lines. Metaphase spreads of CR150 (left) and CR200 (clone II; right) cells were subjected to FISH analysis with an *Abcc4* BAC probe (green) and a *DnajC3* BAC probe (red). Chromosomes were counterstained with DAPI. Colocalization of both probes in amplified regions is highlighted with white circles; green arrow indicates Chr 14 with *Abcc4* and *DnajC3* copies.

As *DnajC3* is located close to *Abcc4* at Chromosome 14qE4, and because we already detected DnajC3 protein overexpression in the fully ciprofloxacin-resistant cells in an ongoing proteomic study [Caceres *et al.*, in preparation], we looked for a possible co-amplification of *DnajC3* with *Abcc4*. FISH analysis with the *DnajC3* specific probe combined with the control probe on Chromosome 14 indeed revealed similar patterns of gene amplification in all the resistant cell lines, like those observed with *Abcc4* probe (data not shown). Moreover, when we hybridized together *Abcc4* and *DnajC3* probes, we observed the co-localization of both probes in amplified regions, either at Chromosome 14 in intermediate resistant cells (CR150 cell line, Figure 4, left panel), or in the hsr in the fully ciprofloxacin-resistant CR200 cell line (Figure 4, right panel).

### Influence of ABCC4 on ciprofloxacin accumulation in murine and human cells

As all the work presented so far had been performed with a murine cell line, we examined whether ciprofloxacin is also a substrate of the human homologue transporter. To this effect, we compared the accumulation of ciprofloxacin (a) in wild-type J774 macrophages (basal expression of Abcc4) and in J774 macrophages resistant to 200 µM of ciprofloxacin and (b) in HEK293 human cells and HEK293/4.63 transduced with the cDNA coding for human ABCC4 and expressing it to high levels [28]. The accumulation of ciprofloxacin was drastically reduced in HEK293/4.63 cells compared to HEK293 cells, and was increased by gemfibrozil, a broad-spectrum inhibitor of MRP transporters (see Figure S3), demonstrating that ciprofloxacin is also a substrate of the human homologue of Abcc4.

## Discussion

The present study shows that an antibiotic is capable of inducing at large concentrations a series of clonal chromosomal alterations in an eukaryotic cell, leading, among other changes, to the overexpression of a multidrug transporter responsible for its efflux. This effect is slowly and only partially reversible at the genomic level. The demonstration remains so far limited to ciprofloxacin and the murine multidrug transporter Abcc4 (Mrp4). However, we show here that ciprofloxacin is also substrate for ABCC4, the human homologue of this transporter (as also found by others [29]), suggesting that similar mechanisms could possibly take place in humans. This opens interesting perspectives in terms of interactions of antibacterial agents with the host in relation to potential cell toxicity, and, in a broader context, in the manner eukaryotic cells deal with exogenous compounds.

*ABCC4* gene amplification as an hsr has already been described in a human T lymphoid cell line made resistant to the antiviral drug adefovir [20], another well known ABCC4 substrate. In this case, however, amplification was located at the distal end of chromosome arm 13q, which is compatible to the normal location of human *ABCC4* (13q32). Hsr and double minute chromosomes are the two common patterns of gene amplification observed upon selection by anticancer agents [6]. Interestingly, we do not observe double minute chromosomes at any step of the selection, in contrast with what has been shown for *Abcb1* (P-glycoprotein) in colchicine-resistant J774.2 cells [30], or for *ABCG2* in mitoxantrone-resistant glioblastoma cells [31], which display double minutes at low drug concentrations, but hsr at high concentrations. In other instances, both patterns are present at the same time, as described for *ABCC1* in a doxorubicin-resistant human tumor cell line [32].

We showed that the karyotypes of the fully resistant clones mainly differ from the one of the wild-type cell line by 3 different marker Chromosomes with *Abcc4* high level amplification. These observations suggest a random chromosomal insertion of the 14qE4 genomic amplification as an hsr, which provides a proliferative advantage by enabling cells to resist to the strong pressure of selection and leading to clonal expansions. Of interest, gene amplification was not restricted to *Abcc4*, since *DnajC3*, another gene located at 14qE4, was co-amplified, and the corresponding protein overexpressed [Caceres *et al.*, in preparation]. Since both *Abcc4* and *DnajC3* genes are constitutively expressed in the wild-type macrophages, they do not need another genetic event in addition to genomic amplification for overexpression, such as a “switch on” induced by a promoter capture or by insertion of retroviral sequences [33], [34]. We did not observe extrachromosomal elements, such as double minutes (dmin) or episomes that are classically reported [6]. However, we cannot exclude their occurrence at the first steps of selection before integration of the amplicons within chromosomes detected in CR200 cells. In cancer cells, genomic amplification has been shown to occur at common chromosomal fragile sites, or to result from defects in DNA replication or telomere dysfunction. It is noteworthy to mention that the chromosomal band 14qE4, with Abcc4 and DnajC3 loci, has been shown to be a common fragile site that may favor genomic instability [35].

The phenotypic reversion is associated with a drastic regression of the *Abcc4* amplification, with loss of the hsr-like amplification of *Abcc4* to Chr 5 and to Chr 16. The persistence of a copy of a marker Chromosome (Chr M, with *Abcc4* hsr) in nearly 30% of the revertant cells, and the time required to obtain phenotypic reversion, underline the stability of the gene amplification. As the revertant cells display a similar phenotype as wild-type cells regarding ciprofloxacin accumulation [27] and almost similar levels of *Abcc4* mRNA [21], it is likely that the *Abcc4* amplification observed on this marker in single copy is not associated with efficient transcription.

*In vitro*, gene amplification is likely to be initiated by a DNA double-strand break in cells that lack appropriate cell-cycle checkpoints [6]. Chemotherapeutic drugs targeting topoisomerase II promote DNA double-strand break, favoring thereby the development of therapy-related leukaemias. In bacteria, fluoroquinolones form a reversible ternary complex linking together DNA and prokaryotic type II topoisomerase enzymes to impair the progression of the replication fork. This leads to several lethal damages, including SOS response induction and, possibly chromosome fragmentation, which explains their rapid bactericidal activity [36]. While no genotoxicity has been reported so far for ciprofloxacin *in vivo* or in cultured cells [37]–[39], chromosomal aberrations have been described in cultured human lymphocytes exposed to supratherapeutic concentrations similar to those used in this study [40], [41], as well as an increase of sister-chromatid exchange [42] and in DNA single strand breaks frequencies [43] in mouse bone marrow cells. Although the concentrations needed to observe these alterations (100–200 µM, i.e. 40–80 mg/L) are well above the serum levels observed in patients receiving conventional therapies (1–4 mg/L), one needs to take into consideration that ciprofloxacin accumulates in tissues (with tissular levels reaching values 2–7 fold higher than serum levels) as well as in body fluids, with the highest concentrations (200–900 mg/L) being found in urine. This suggests that *in vivo* exposure may be more important than anticipated based on serum levels only.

Although pending for further investigations aimed at elucidating the mechanism leading to gene amplification, our data thus indicate that ciprofloxacin may induce in eukaryotic cells chromosomal aberrations leading to overexpression of the transporter responsible for its efflux. Whether such alterations may occur *in vivo* will clearly depend on the concentration of the drug, and probably also on a combination of its recognition by efflux transporters and its capacity to interact with DNA-topoisomerase complexes. Other fluoroquinolones may, indeed, be much more toxic than ciprofloxacin in this context [41], [44], with many of them having been withdrawn or not accepted for registration in many countries for unsatisfactory benefit to risk ratio involving, among other untoward reactions, clastogenic effects (see [45] for an example with gemifloxacin). It is interesting to note that N-substituted piperazinyl quinolones derived from ciprofloxacin or other clinically-used fluoroquinolones show *in vitro* cytotoxicities that are as high as those seen with etoposide, a well known inhibitor of topoisomerase II [46], [47]. These compounds are now evaluated as potential anticancer agents.

## Materials and Methods

### Chemicals

All cell culture reagents were purchased from Invitrogen (Carlsbad, CA). Ciprofloxacin HCl (potency 85%) was received from Bayer HealthCare (Wuppertal, Germany) as microbiological standard. Other chemical products were purchased from Sigma-Aldrich (St. Louis, MO).

### Cell lines and culture conditions

J774 mouse macrophage-like cells [48] (referred to as wild-type cells) were cultured and maintained as already described [49]. Ciprofloxacin-resistant macrophages and their revertant were fully described in a previous publication of our group [27]. Resistant cells were obtained by chronic exposure to progressively increasing concentrations of ciprofloxacin (100 µM, 150 µM, and 200), yielding to cell lines referred to here as CR100 (used at the 5th passage), CR150 (used at the 3rd passage) and CR200 (used at the 76th passage) respectively. Revertant cells (referred to here as Rev200) were obtained by returning CR200 cells to ciprofloxacin-free and used here at their 84th passage in the absence of selective pressure. Human Embryonic Kidney HEK293 cells and HEK293/4.63 cells transduced with human *ABCC4* cDNA were obtained from P. Borst (*Het Netherlands Kanker Instituut*, Amsterdam, The Netherlands). They were cultivated in Dulbecco's modified Eagle's medium supplemented with 10% fetal calf serum [28].

### Accumulation and efflux of fluoroquinolones

These experiments were performed exactly as previously described [21], [27], [50]. Cell-associated ciprofloxacin was assayed by fluorimetry (λ_exc_ = 275 nm and λ_em_ = 450 nm) and its cellular concentration expressed by reference to the total cell protein content as measured by the Lowry's method [51].

### Quantification of *Abcc4* by real-time PCR and Western blot Analysis

*Abcc4* expression was assessed at mRNA and proteins levels, as described previously [21]. Real-time PCR experiments were performed starting from 1 µg of total purified RNA transcribed into cDNA, and using SYBR Green detection. Two housekeeping genes, *Ywhaz* and *Rpl13a* (mouse geNorm normalization kit, PrimerDesign Ltd., Southampton, UK) were used for normalization. The relative quantification of *Abcc4* gene in cell lines of interest was done using levels measured for wild-type J774 macrophages as baseline, based on Pfaffl's equation [52]. Western-blots were performed on cell crude extracts. After electrophoresis on acrylamide gel and transfer to nitrocellulose membrane, proteins of interest were detected using anti-ABCC4 monoclonal antibody (M_4_I-10; Alexis Biochemicals, Lausen, Switzerland) or anti-actin polyclonal antibodies (Sigma-Aldrich) (dilution 1/1000), followed by appropriate horseradish peroxidase-coupled secondary antibodies (dilutions 1/600). Blots were then revealed by chemiluminescence assay (SuperSignal West Pico, Pierce, Thermo Fisher Scientific Inc., Rockford, IL).

### Cytogenetic analysis

Metaphase chromosomes were obtained according to standard methods for mouse cell lines [53]. Cells were grown for 48 h into 6-well plates (initial density of 5×10**^4^** cells per cm**^2^**) and fed with fresh medium 4 h before addition of a mitotic spindle inhibitor (KaryoMAX**®** Colcemid**™** Solution, Invitrogen) at a final concentration of 0.02 µg/ml. After 45 min of incubation at 37°C, cells were washed with PBS, detached by trypsinization and collected by centrifugation (900 rpm, 10 min). They were then submitted to hypotonic choc by resuspension in KCl 75 mM and incubation for 10 min at room temperature. After centrifugation (900 rpm, 5 min), cell pellets were resuspended in Carnoy's fixative (methanol/glacial acetic acid, 3/1) and incubated 15 min at room temperature. This fixation step was repeated three times. Metaphase chromosomes were obtained after spreading and air-drying of fixed cells onto microscope glass slides. Metaphase cells were banded with trypsin denaturation followed by a Wright staining. Metaphases were analysed with Ikaros Imaging System (MetaSystems, Altlussheim, Germany). Karyotypes were described according to The Rules for Nomenclature of Chromosome Aberrations (Revised: January 2005) from the International Committee on Standardized Genetic Nomenclature for Mice (http://www.informatics.jax.org/mgihome/nomen/anomalies.shtml). A total of 8 and 21 metaphasic cells were analyzed, for wild-type and resistant macrophage (CR200) cell lines, respectively.

### Fluorescence in situ hybridization analysis

Fluorescence in situ hybridization (FISH) analysis was performed using Bacterial Artificial Chromosome (BAC) probes (from the Roswell Park Cancer Institute, Buffalo, NY) specific for *Abcc4* (clone RP23-390O15) and *DnajC3* loci (clone RP23-378H12) (both located at Chr 14qE4), and for a more centromeric locus of Chromosome 14 which served as control (clone RP23-364J13, located at Chr 14qA3). BAC localizations have been checked by end sequencing analysis on a Beckman CEQ2000 sequencer (Beckman Coulter, Fullerton, CA). After extraction, BAC probes were labeled by random priming with fluorescent dUTP (Cy3-dUTP [red] or fluorescein-12-dUTP [green]), using the BioPrime**®** DNA Labeling System (Invitrogen). Hybridization and fluorescence detection were carried out according to standard procedures [54]. Briefly, fluorescent probes (*Abcc4* or *DnajC3* probes: 20 ng/µl; control probe: 24 ng/µl), denatured at 70°C for 5 min and preannealed with a 50-fold excess of mouse Cot-1 DNA (Invitrogen), were hybridized overnight at 37°C to cytogenetic slides pretreated with pepsin 0.01%/HCl 0.01 M at 37°C for 13 min, and then denatured at 75°C for 2 min 20 sec in 70% formamide/2xSSCP (saline sodium citrate phosphate buffer). After washing (2 min in 0.4xSSC [saline sodium citrate buffer] at 30°C), cells were counterstained with 4,6-diamidino-2-phenylindole (DAPI) and observed with a Zeiss Axioplan2 microscope equipped with a HBO103W lamp (Zeiss, Oberkochen, Germany). Images were captured with the Isis Imaging System (MetaSystems). For each hybridization, a minimum of 30 metaphases and of 200 interphasic cells were analyzed.

### Multicolour FISH (mFISH)

Fresh metaphase spreads were pretreated with RNase A (100 µg/ml)/2xSSC for 45 min at 37°C, then denatured as previously described. The multicolour probe kit (21XMouse, MetaSystems) was denatured according to manufacturer's instructions, and then hybridized to metaphases at 37°C for 3 to 4 days. Post-hybridization washes and counterstaining were performed according to manufacturer's instructions, using DAPI/antifade (MetaSystems). Ten metaphases were analyzed for wild-type macrophage cells, and more than 20 for resistant cells CR200. When mFISH was combined with *Abcc4* probe hybridization, we compared metaphases hybridized first with mFISH probes, then washed and hybridized again with the *Abcc4* probe.

## Supporting Information

Figure S1 **mFISH karyotype of wild-type J774 macrophages.** Chromosomes are displayed with false colors, as indicated by rounds; squares indicate the combination of true colors given by the probes.
(TIF)

Figure S2 ***Abcc4*** **FISH and mFISH analysis in wild-type J774 macrophages, in cells resistant to increasing concentrations of ciprofloxacin, and in revertant cells.** A: Metaphase spreads of J774 wild-type (upper left panel), CR100 (upper right panel), CR150 (middle panel) and Rev200 (lower panel) cells were subjected to FISH analysis with an *Abcc4* BAC probe (green) and a control BAC probe located on Chr 14 (red). Chromosomes were counterstained with DAPI. Representative metaphases of the different clones observed are shown. Green arrow indicates Chr 14 with *Abcc4* copy, red arrow points to the control BAC probe (red) located on Chr 14, green circle indicates *Abcc4* amplification, and green square *Abcc4* additional copy. B: Metaphase spreads of the main three clones (I, II, III) observed in CR200 cells hybridized first with mFISH probes and subsequently with the *Abcc4* BAC probe (green) and the control BAC probe (red) located on Chr 14 (chromosomes counterstained with DAPI).
(TIF)

Figure S3 **Comparison of ciprofloxacin accumulation in murine and human cells with basal or overexpression of Abcc4/ABCC4.** Cellular accumulation of ciprofloxacin in J774 mouse macrophages (WT or CR200) and in human embryonic kidney cells (HEK293, parental cells; HEK293/4.63 transduced with the human cDNA coding for ABCC4 and overexpessing the transporter to high levels [28], [29]). Cells were incubated during 2 h with an extracellular concentration of 20 mg/L (50 µM) of ciprofloxacin in the absence of in the presence of 500 µM gemfibrozil. Data are expressed in percentage of the value measured in control condition in the parental cell line and are the mean ± SD of 3 independent determinations.
(TIF)
