# Supplementary figures and images for: Characterization of Abcc4 Gene Amplification in Stepwise-Selected Mouse J774 Macrophages Resistant to the Topoisomerase II Inhibitor Ciprofloxacin
Source: PLoS One. 2011 Dec 5;6(12):e28368. doi: 10.1371/journal.pone.0028368 (PMC3230599; doi:10.1371/journal.pone.0028368)

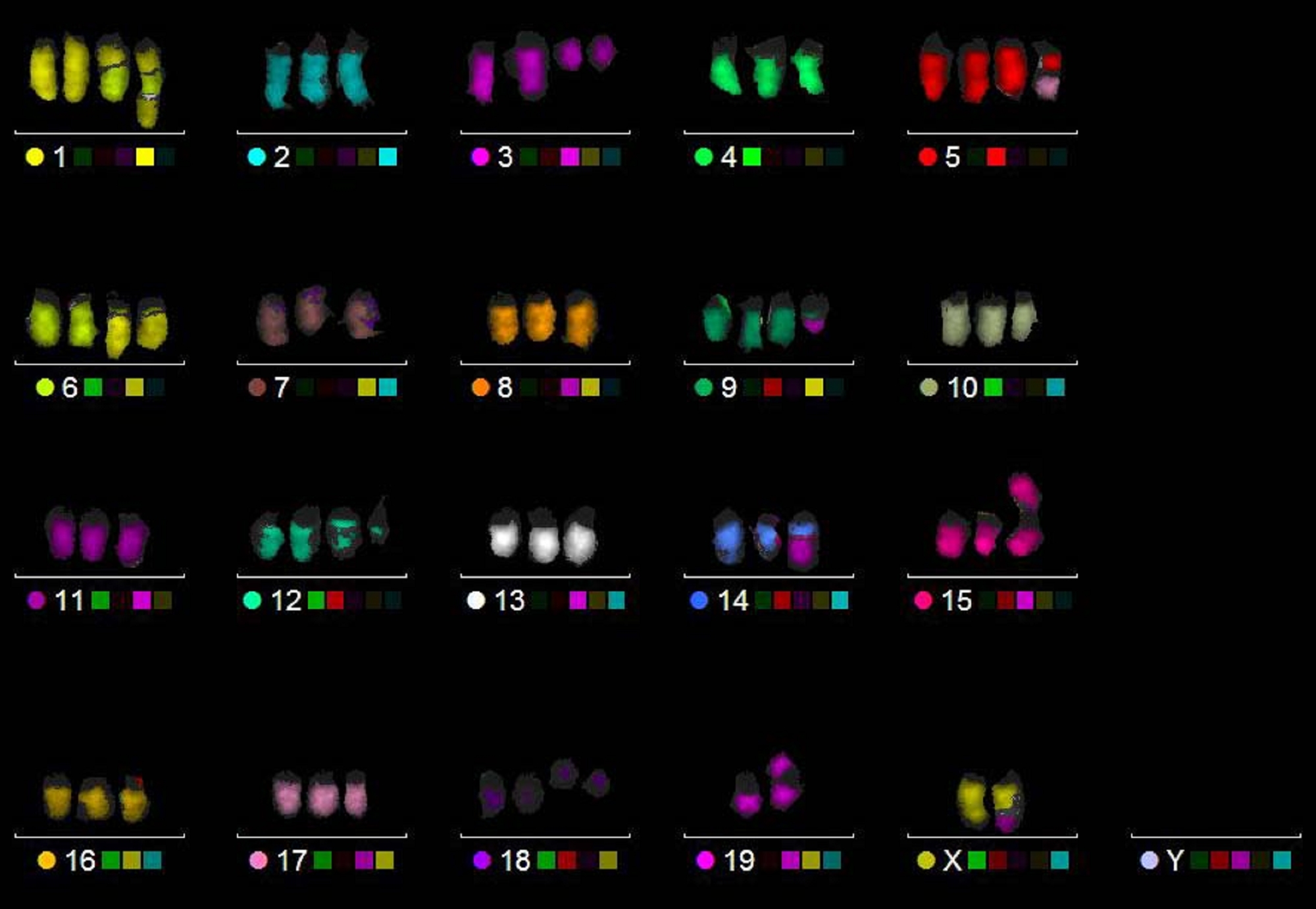

Supplement: Figure S1 — mFISH karyotype of wild-type J774 macrophages. Chromosomes are displayed with false colors, as indicated by rounds; squares indicate the combination of true colors given by the probes. (TIF) [file pone.0028368.s001.tif]

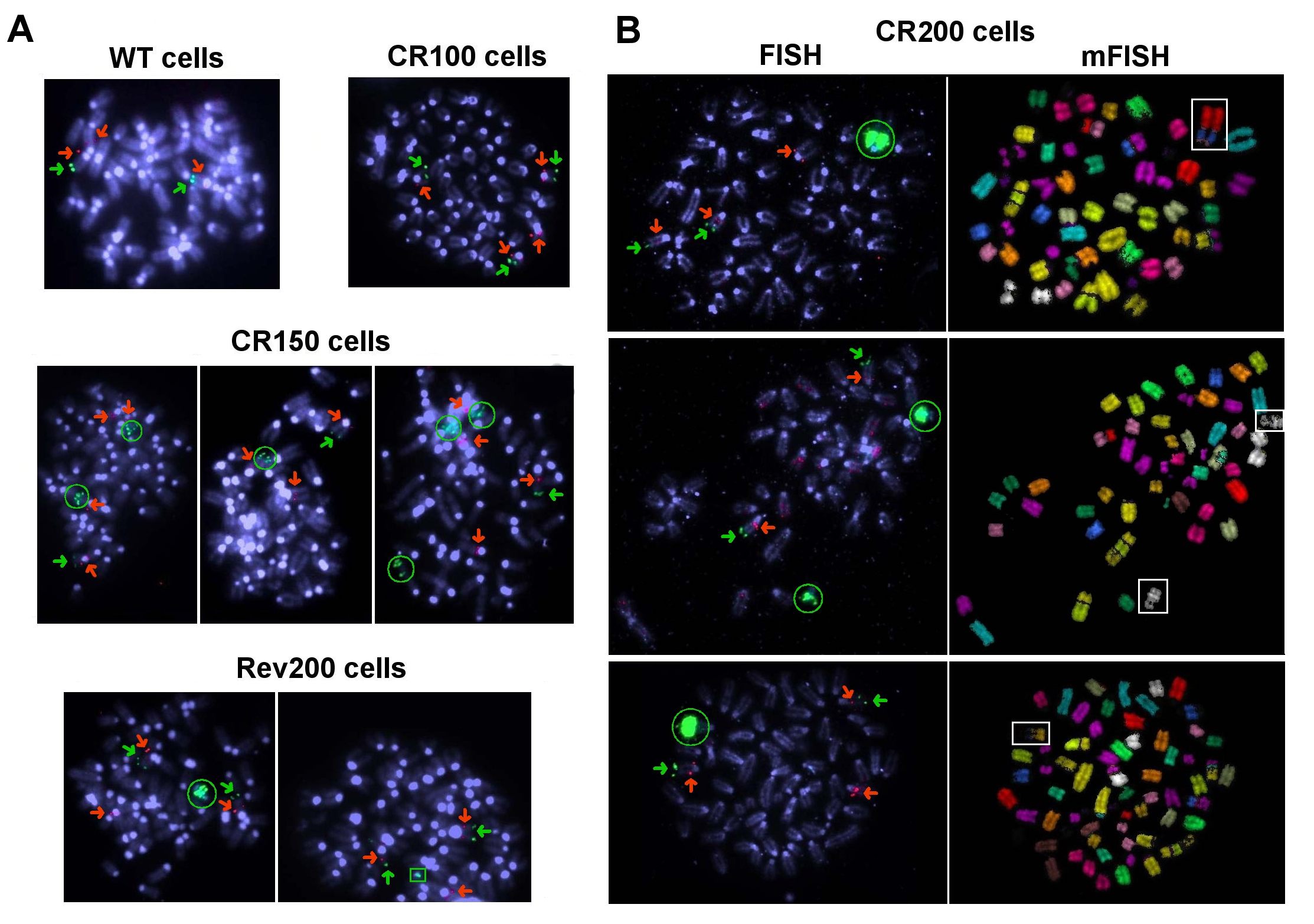

Supplement: Figure S2 — Abcc4 FISH and mFISH analysis in wild-type J774 macrophages, in cells resistant to increasing concentrations of ciprofloxacin, and in revertant cells. A: Metaphase spreads of J774 wild-type (upper left panel), CR100 (upper right panel), CR150 (middle panel) and Rev200 (lower panel) cells were subjected to FISH analysis with an Abcc4 BAC probe (green) and a control BAC probe located on Chr 14 (red). Chromosomes were counterstained with DAPI. Representative metaphases of the different clones observed are shown. Green arrow indicates Chr 14 with Abcc4 copy, red arrow points to the control BAC probe (red) located on Chr 14, green circle indicates Abcc4 amplification, and green square Abcc4 additional copy. B: Metaphase spreads of the main three clones (I, II, III) observed in CR200 cells hybridized first with mFISH probes and subsequently with the Abcc4 BAC probe (green) and the control BAC probe (red) located on Chr 14 (chromosomes counterstained with DAPI). (TIF) [file pone.0028368.s002.tif]

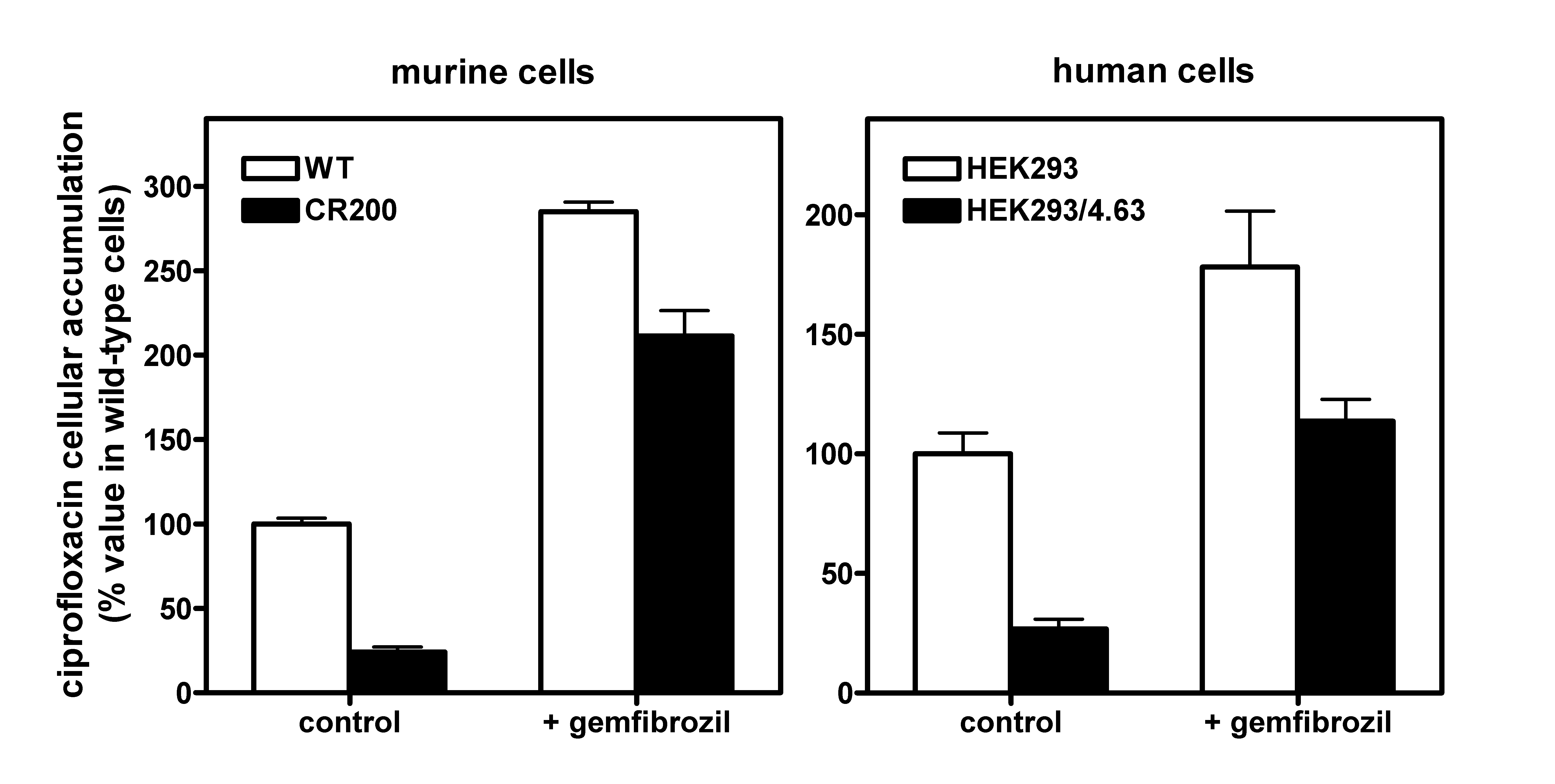

Supplement: Figure S3 — Comparison of ciprofloxacin accumulation in murine and human cells with basal or overexpression of Abcc4/ABCC4. Cellular accumulation of ciprofloxacin in J774 mouse macrophages (WT or CR200) and in human embryonic kidney cells (HEK293, parental cells; HEK293/4.63 transduced with the human cDNA coding for ABCC4 and overexpessing the transporter to high levels [28], [29]). Cells were incubated during 2 h with an extracellular concentration of 20 mg/L (50 µM) of ciprofloxacin in the absence of in the presence of 500 µM gemfibrozil. Data are expressed in percentage of the value measured in control condition in the parental cell line and are the mean ± SD of 3 independent determinations. (TIF) [file pone.0028368.s003.tif]
